# The global epidemiology of gastrointestinal cancers attributable to high-BMI: findings from the Global Burden of Disease Study 2021

**DOI:** 10.3389/fnut.2025.1670111

**Published:** 2025-09-23

**Authors:** Laiang Yao, Tao Ji, Hailian Mu, Xiangming Xu

**Affiliations:** Department of Gastroenterology, Linyi People’s Hospital, Linyi, China

**Keywords:** gastrointestinal cancer, high-BMI, cancer epidemiology, Global Burden of Disease, overweight and obesity

## Abstract

**Background:**

Gastrointestinal (GI) cancers collectively account for over 30% of global cancer-related mortalities. Obesity is a well-established risk factor for GI cancers. We aim to investigate the temporal trends of GI cancer attributable to high BMI between 1990 to 2021 on global, regional and national levels.

**Methods:**

Data was obtained from the Global Burden of Disease Study 2021. Deaths, Disability-adjusted life years (DALYs), and age-standardized rates (ASRs) were used to measure disease burden. Estimated annualized percent change (EAPC) were used to assess the temporal trend. We explored the association between SDI with high BMI-attributed GI cancer burden across 21 regions, 204 countries over the three decades.

**Results:**

Globally, between 1990 and 2021, all GI cancers had rising trends of deaths and DALYs attributable to high BMI. Colorectal cancer constituted the largest disease burden, with 99,268 (95% UI: 42,596–157,948) deaths and 1.17 (95% UI: 0.51–1.87) million DALYs in 2021. Liver cancer and pancreatic cancer had the sharpest increase in age-standardized rates, with EAPC exceeding 4 and 2%, respectively. Substantial heterogeneity was observed among the disease burden and temporal trends across regions. Higher SDI countries reported higher ASRs of GI cancers but showed declining trends with risk-attributable colorectal cancer, gallbladder and biliary tract cancer. Nearly all regions had increasing trends with the ASRs of high-BMI caused liver cancer and pancreatic cancer.

**Conclusion:**

The global burden of gastrointestinal cancers attributable to high BMI has increased substantially over the past three decades. We observed significant disparity with the temporal trends of different gastrointestinal cancers at various regions. Multi-level interventions, including lifestyle modification and tailored screening practice is required at global and regional levels to address the burden of gastrointestinal cancers attributable to high BMI.

## Background

Gastrointestinal (GI) cancers are malignancies originating in the gastrointestinal tract, including the esophagus, stomach, liver, pancreas, gallbladder, small intestine, colon, rectum and anus (1). Global estimates have indicated that GI cancers collectively comprise over one-fourth of all cancer incidences and more than one-third of all cancer-related mortalities (2, 3). A previous population-based systematic analysis indicated the lifetime risk of the development and mortality of GI cancers was 8.2 and 6.2%, respectively (4). Notably, five GI cancers ranked among the 10 deadliest cancers globally as of 2022, reflecting their particularly poor prognosis (3). Despite advancements in therapeutic strategies, projections suggest an escalating disease burden of GI cancers in the coming decades, underscoring the urgent need for improved preventive and management approaches (5, 6).

There are several shared risk factors for GI cancers, with obesity being a well-established risk factor (7, 8). Previous meta-analysis and pooled analysis revealed that high-BMI was associated with an increased risk of colorectal cancers, stomach cancers and hepatocellular cancers by 40, 20, and 90% respectively, compared to normal BMI (body mass index) individuals (9–11). The prevalence of obesity has risen in all world regions over the past three decades, with 43% of adults being overweight and 16% of adults living with obesity in 2022 (12). In addition, with the combined effect of sedentary lifestyles and consumption of processed and unhealthy foods, the prevalence of obesity is likely to continue to increase globally (13).

It is evident that the epidemiological patterns of GI cancers and obesity-related risks exhibit substantial heterogeneity across geographic regions, demographic groups, and organ sites. Variations in socioeconomic development, healthcare access, and lifestyle factors further complicate this relationship. Leveraging the most recent and comprehensive data from the Global Burden of Disease (GBD) Study 2021, which offers the widest scope of global epidemiological data, our study provides a novel and systematic investigation into the high-BMI attributable burden of gastrointestinal (GI) cancers. While previous studies, including earlier GBD analyses, have primarily focused on the general epidemiology and overall disease burden of GI cancers, our work specifically quantifies the risk-attributable burden by assessing the temporal trends, geographical and demographic disparities. We aim to provide critical insights for targeted public health initiatives aiming at mitigating the growing impact of obesity on GI cancer burden worldwide.

## Methods

### Data source

Data was obtained from the Global Burden of Disease Study 2021, which is a systematic effort to estimate the burden of 371 disease and 88 risk factors across 204 countries and territories from 1990 to 2021 (14, 15). All data regarding the disease burden of GI cancers attributable to high-BMI was publicly available through the online query tool Global Health Data Exchange (https://vizhub.healthdata.org/gbd-results/). Our study followed the Guidelines for Accurate and Transparent Health Estimates Reporting Guidelines for cross-sectional studies (GATHER) (16).

### Definitions

The detailed data sources, estimation methodology, and risk-attributed burden of the GBD 2021 has been previously published (15). In brief, high BMI was defined as BMI, calculated as dividing an individual’s weight in kilograms by the square of their height in meters (kg/m^2^) greater than 25 kg/m^2^ for adults aged 20 years and older (15, 17). Representative studies reporting data with overweight and obesity prevalence, or mean BMI was included to ascertain the exposure. In our study, we used deaths and DALYs (Disability-adjusted life years) to measure disease burden. Mortality data was primarily obtained from population-based cancer registries, vital registration systems and verbal autopsy studies. DALY is a metric that sums the years of life lost with premature mortality and years of life with disability, with each DALY representing 1 year of loss of optimal health. A comparative risk assessment method was leveraged to quantify the burden of GI cancers attributable to high BMI. Briefly, the exposure and distribution of risk factors are estimated by Bayesian meta-regression modeling and spatiotemporal Gaussian process methods to calculate the relative risk (RR) for each risk-outcome pair. The attributable disease burden was based on the concept of the theoretical minimum risk exposure level (TMREL), which represents the level of risk exposure that minimizes the risk at population levels. The disease burden is multiplied by the deaths and DALYs with the population attributable fractions (PAFs), whereas PAF indicates the proportion of risk that would be reduced if BMI was reduced to TMREL. In our study, four GI cancers were identified with high-BMI attributed disease burden, which are colon and rectum cancers (CRC), gallbladder and biliary tract cancers (GBTC), liver cancers and pancreatic cancers. We observed negative estimates of the death and DALY rates of pancreatic cancer attributable to high-BMI among certain Asian and African regions. This is likely due to the effect modification and differences in relative risks of the high BMI and pancreatic cancer pair, which was primarily derived from data of Western countries and regions. In addition, for high BMI, the TMREL is established within the normal weight range (e.g., 21–23 kg/m^2^). The negative estimates may indicate that the prevalent BMI distribution was lower than the TMREL on average; consequently, the model calculates that a shift toward the TMREL would result in an increase in disease burden, not a reduction. The Socio-demographic index (SDI) is a composite measure derived from three indicators: the total fertility rate among women under 15, educational attainment for individuals aged 15 and above and per capita income adjusted for time lag. The index ranges from 0 (indicating low development) to 1 (indicating high development), categorizes countries and territories into five quintiles, from low to high developmental status, and provides an essential understanding of the association between socioeconomic factors and various health outcomes.

### Statistical analysis

We explored the disease burden of GI cancers attributable to high BMI across various demographics, including age, sex, temporal trends and geographical locations. The total number of deaths and DALYs, along with the age-standardized rates (ASRs) were reported with the 95% uncertainty intervals (95% UI). ASRs were calculated using the director method based on population estimates. 95% UIs were calculated as the 2.5th and 97.5th ranked values from a posterior distribution of 1,000 draws. We assessed the temporal trend of GI cancers attributable to high-BMI from 1990 to 2021 with estimated annualized percent changes (EAPCs) of the ASRs. EAPC were estimated using log-linear regressions, with assumptions of the natural log of rate following a Poisson distribution, expressed as $\ln_{\text{rate}}=\alpha +\beta_{\text{year}}+\varepsilon$ . The Beta-coefficient represents the positive or negative trends of the ASRs. EAPCs and their 95% confidence intervals were calculated with the formula $100\times (\exp_{\beta }-1)$ . ASRs were considered to have an increasing trend with the lower limit of the 95% confidence interval of EAPCs were greater than 0 while ASRs were considered to have a decreasing trend with the upper limit of the 95% CI of EAPC to be less than 0. Further, we investigated the association between SDI with the disease burden of GI cancers attributable to dietary risks across different locations and years. We performed all data analysis, visualizations with RStudio (v.2024.04.2).

## Results

**Figure 1:**
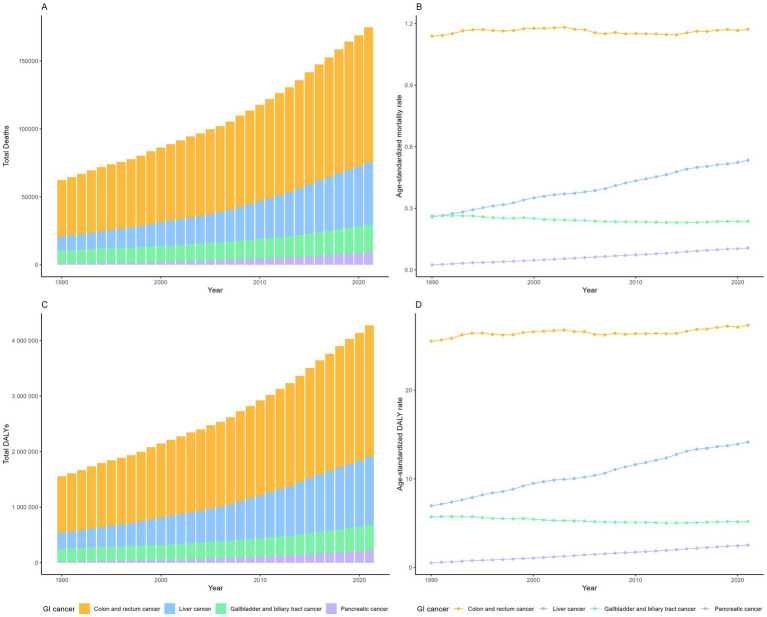
The number of deaths **(A)** and DALYs **(C)** of gastrointestinal cancers attributable to high BMI globally, from 1990 to 2021; The age-standardized mortality rate **(B)** and age-standardized DALY rate **(D)** of gastrointestinal cancers attributable to high BMI globally.

In 2021, there was an estimated burden of 99 thousand deaths and 2.36 million DALYs of CRC, 46 thousand deaths and 1.24 million DALYs of liver cancer, 20 thousand deaths and 0.45 million DALYs of GBTC, 9 thousand deaths and 0.22 million DALYs of pancreatic cancer attributable to high BMI globally (Table 1; Figure 1). All GI cancers showed rising trends with the total deaths and DALYs since 1990, with pancreatic cancer showing the largest increase (Table 1; Figure 1). The four GI cancers showed different changes with the ASRs between 1990 and 2021. With no significant changes of the EAPC in ASMR (0.00, −0.04 to 0.04%) and a slight increase of the ASDRs (EAPC: 0.12, 0.08–0.16%), CRC remained the highest ASRs over the period. Pancreatic cancer had the lowest ASRs globally, but witnessed the most rapid increase, with the EAPCs of ASMR and ASDR 4.63% (4.42–4.84%) and 4.84% (4.6–5.09%) respectively. There was also an significant increase of the ASRs of high-BMI attributed liver cancer globally, with the EAPCs exceeding 2% globally. On the other hand, gallbladder and biliary tract cancer was the only GI cancer that showed a declining trend globally (EAPC: −0.46% [−0.53 to −0.38%] in ASMR and −0.44% [−0.52% to −0.37%] in ASDR). By 2021, 9.65% of CRC age-standardized DALYs, 12.05% of GBTCs age-standardized DALYs, 9.48% of liver cancer age-standardized DALYs and 1.96% of pancreatic cancers age-standardized DALYs were attributable to high BMI (Supplementary Figure S1).

**Table 1:** Deaths, DALYs and age-standardized rates (ASRs) of gastrointestinal cancers attributable to high-BMI in 1990, 2021 and the estimated annualized percent change (EAPC) from 1990 to 2021.

	1990				2021					
	Deaths	DALY	ASMR	ASDR	Deaths	DALYs	ASMR	ASDR	EAPC of ASMR	EAPC of ASDR
Colorectal cancer	41,536 (17,666, 67,379)	1,015,042 (429,787, 1,631,973)	1.14 (0.48, 1.86)	25.54 (10.83, 41.20)	99,268 (42,956, 157,948)	2,364,664 (1,021,593, 3,752,340)	1.17 (0.51, 1.87)	27.33 (11.80, 43.47)	0.00 (−0.04, 0.04)	0.12 (0.08, 0.16)
Liver cancer	10,282 (4,197, 16,721)	292,696 (119,095, 475,963)	0.26 (0.11, 0.42)	6.97 (2.84, 11.34)	46,203 (18,606, 77,983)	1,237,313 (504,239, 2,101,958)	0.53 (0.21, 0.90)	14.16 (5.77, 24.06)	2.37 (2.29, 2.45)	2.31 (2.22, 2.39)
Pancreatic cancer	955 (−2,011, 5,926)	21,952 (−49,859, 141,075)	0.02 (−0.05, 0.16)	0.55 (−1.24, 3.53)	9,215 (−2,377, 26,607)	223,368 (−47,782, 626,550)	0.11 (−0.03, 0.31)	2.54 (−0.56, 7.16)	4.63 (4.42, 4.84)	4.84 (4.6, 5.09)
Gallbladder and biliary tract cancer	9,680 (6,644, 13,261)	227,609 (156,891, 309,773)	0.26 (0.18, 0.36)	5.74 (3.95, 7.83)	20,102 (13,656, 27,792)	451,489 (308,188, 621,591)	0.24 (0.16, 0.33)	5.20 (3.56, 7.17)	−0.46 (−0.53, −0.38)	−0.44 (−0.52, −0.37)

The significant global increase in deaths and DALYs from high BMI-attributed GI cancers from 1990 to 2021 was consistent in both sexes (Supplementary Figures S2, S3). In 2021, the burden was higher among males for liver (28.5 k vs. 17.7 k deaths; 0.80 M vs. 0.43 M DALYs) and colorectal cancers (51 k vs. 48 k deaths; 1.3 M vs. 1.1 M DALYs). In contrast, females bore a higher burden for gallbladder and biliary tract (7.95 k vs. 6.6 k deaths; 0.27 M vs. 0.18 M DALYs) and pancreatic cancers (5.6 k vs. 3.6 k deaths; 127 k vs. 96 k DALYs). ASRs increased for all GI cancers in males but decreased for colorectal and gallbladder/biliary tract cancers in females (Supplementary Figures S4, S5). Pancreatic cancer exhibited the most substantial rise in ASRs for both sexes.

**Figure 2:**
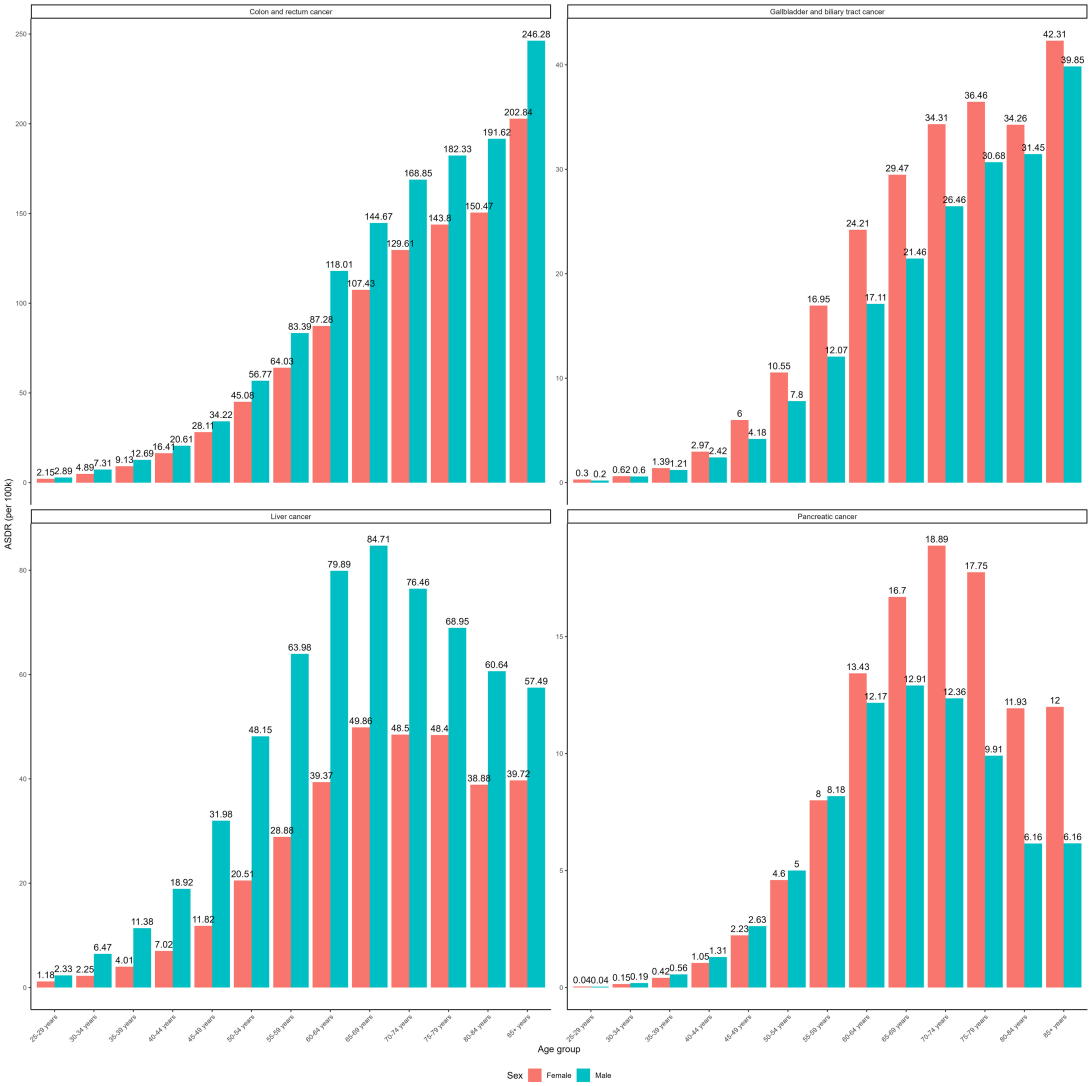
Age-specific DALY rates of gastrointestinal cancers attributable to high BMI between sex in 2021.

The age-specific rates of high BMI-attributable GI cancer burden increased with age in both sex 9 (Figure 2). However, the specific age groups with the highest DALY rates varied by cancer type: 85 years and above for CRC and GBTCs, and 65–74 years for liver and pancreatic cancers. Males demonstrated higher DALY rates across all age groups for CRC and liver cancer, whereas females exhibited higher rates for GBTCs. A sex-specific disparity was observed for pancreatic cancer, with higher rates in males under 60 and in females aged 60 and above. Noteworthy is the substantial gender disparity in liver cancer, with males displaying approximately twice the ASRs compared to females (ASMR: 0.7 vs. 0.38 per 100,000 population; ASDR: 19.05 vs. 9.52 per 100,000 population).

### Colorectal cancer

**Figure 3:**
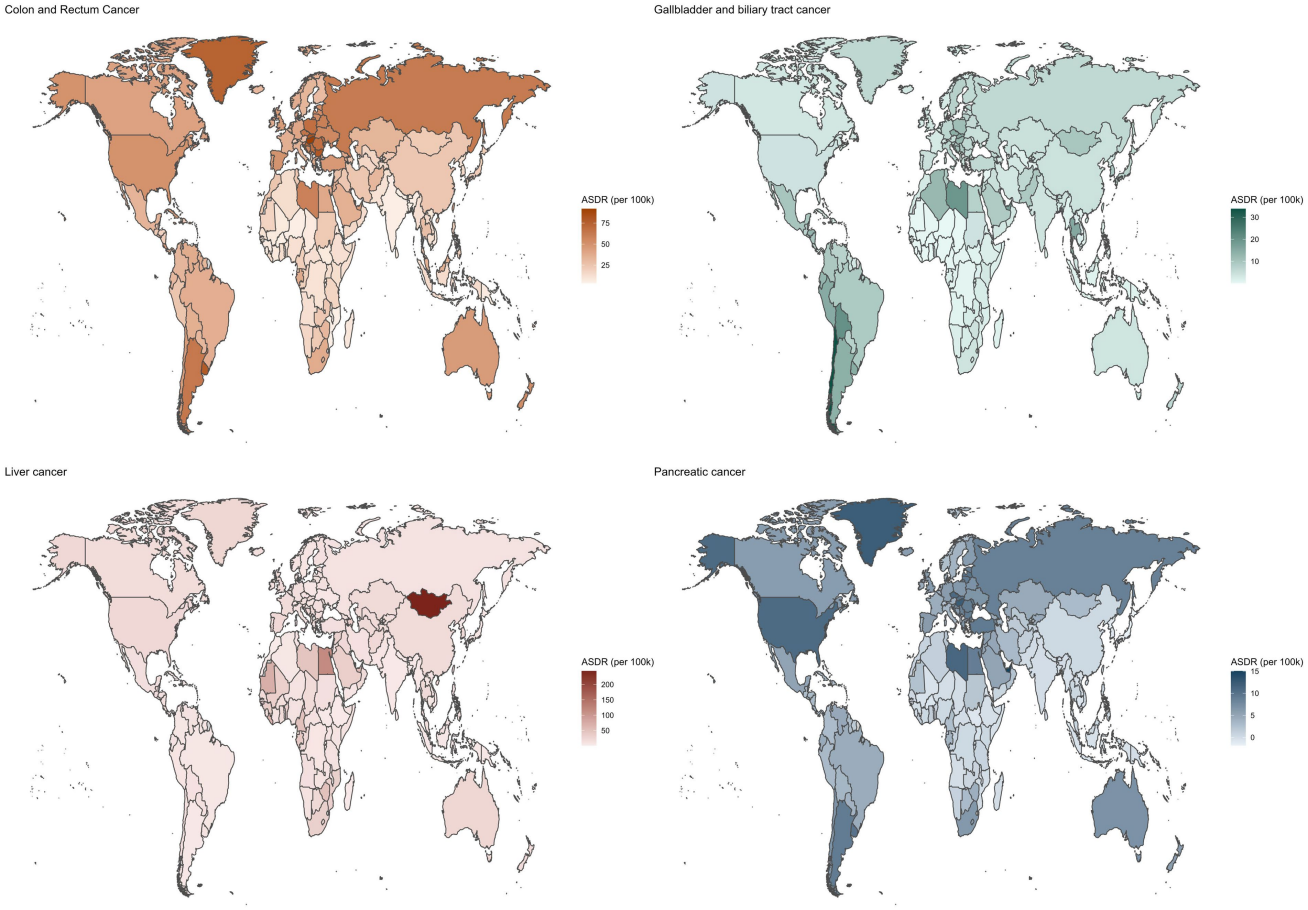
Age-standardized DALY rates of gastrointestinal cancers attributable to high BMI across 204 countries and territories for both sex in 2021.

**Figure 4:**
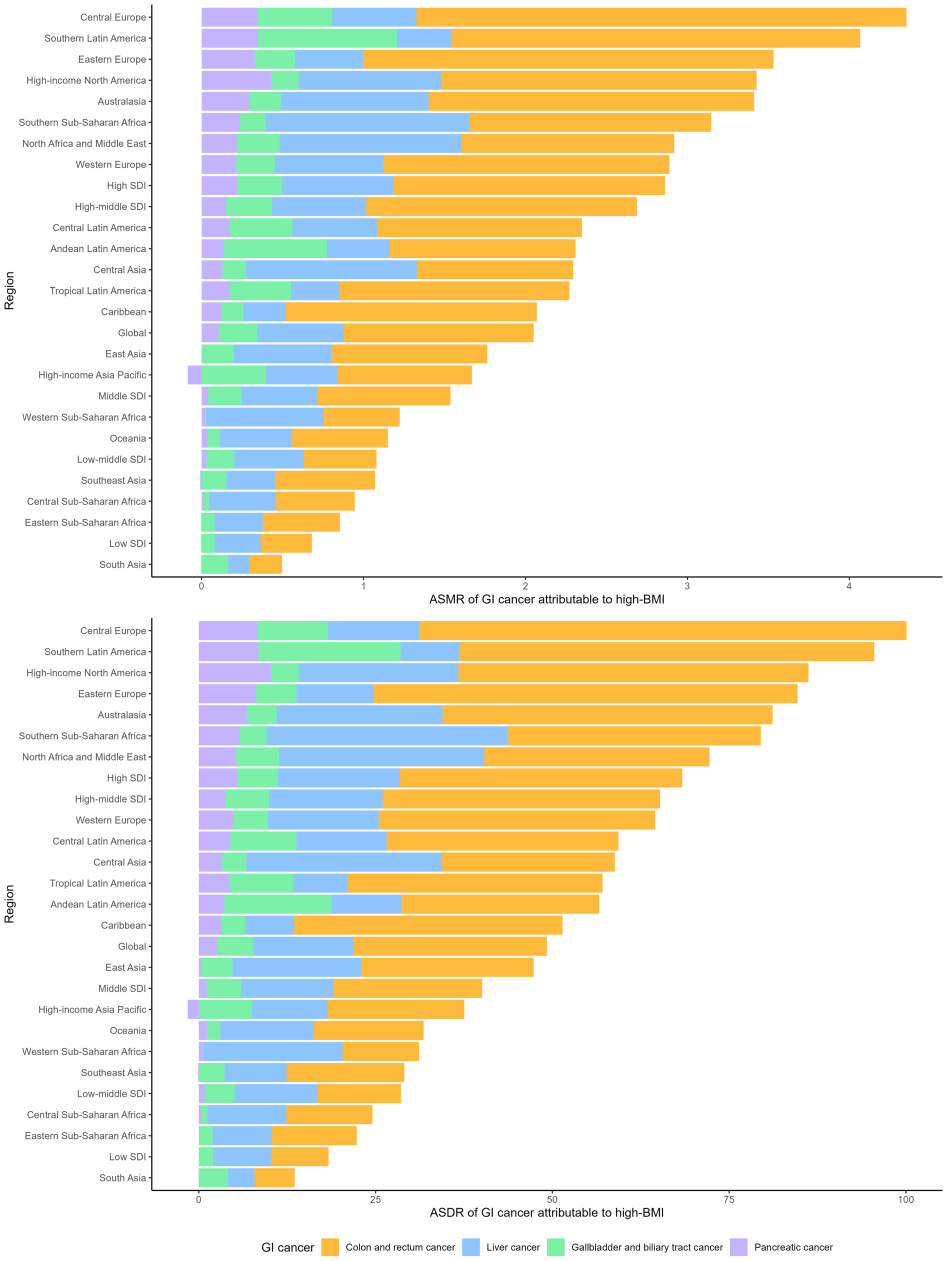
The age-standardized mortality rate and age-standardized DALY rate of gastrointestinal cancers attributable to high BMI across regions in 2021.

**Figure 5:**
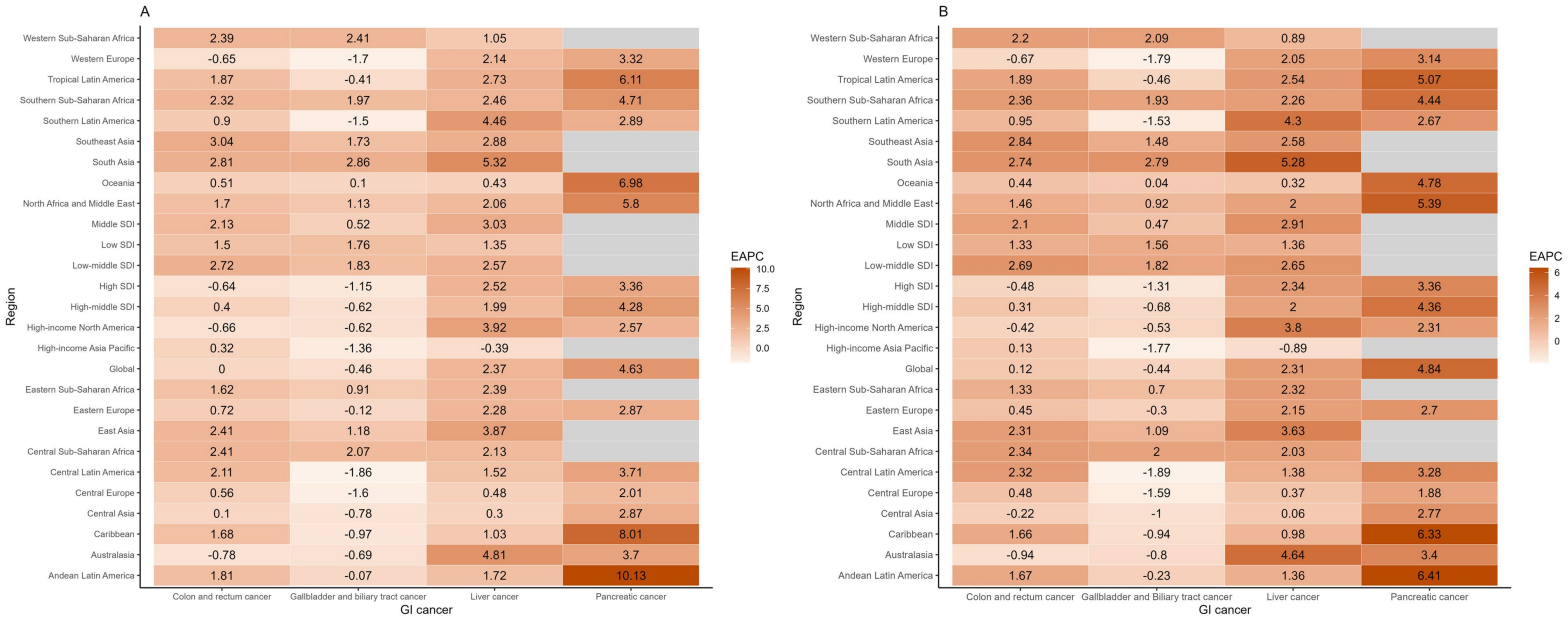
The estimated annual percent changes in ASMR **(A)** and ASDR **(B)** of gastrointestinal cancers attributable to high BMI across GBD regions from 1990 to 2021.

**Figure 6:**
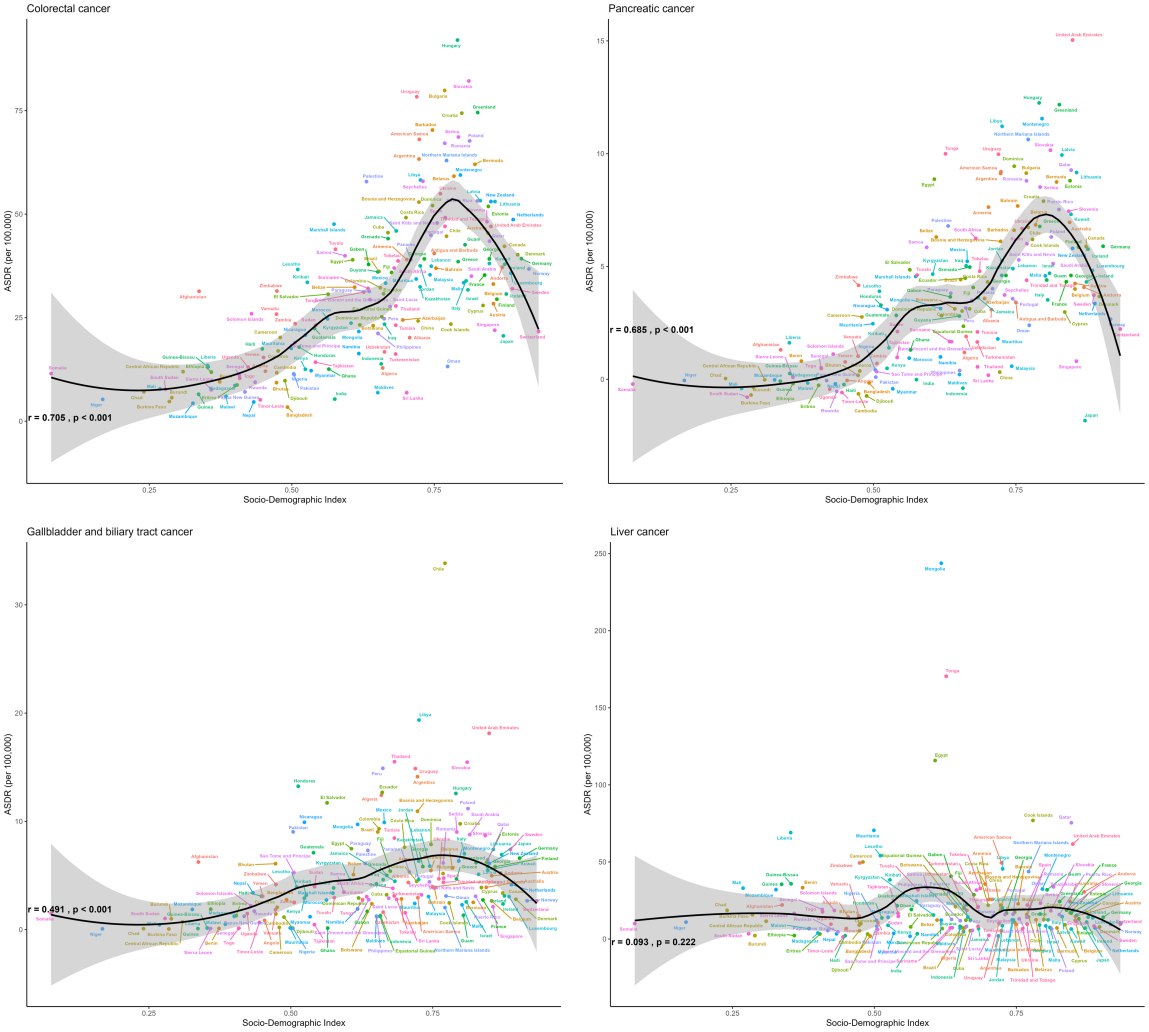
The trends in ASDR of gastrointestinal cancers attributable to high BMI across 21 GBD regions by SDI from 1990 to 2021.

High SDI and high-middle regions had the highest ASRs of high BMI attributed CRCs in 2021, with Central Europe, Eastern Europe and Southern America exhibiting the highest ASRs (ASMR exceeding 2.5 per 100,000 population and ASDR exceeding 50 per 100,000 population) (Figures 3, 4; Supplementary Table S1). Low SDI regions had the lowest ASRs, with the lowest ASRs observed in South Asia. Three high-SDI regions, including Australasia, Western Europe and high-income North America showed significant decline of the ASRs of CRC attributable to high-BMI from 1990 to 2021 (Supplementary Table S1; Figures 5, 6). Southeast Asia experienced the most significant increase, with EAPCs of 3.04% (2.93–3.15%) in ASMR and 2.84% (2.72–2.97%) in ASDR, followed by South Asia, East Asia and Sub-Saharan African regions. High BMI corresponds with the largest fraction of CRC age-standardized DALYs in High-income North America (16%), followed by North Africa and Middle East (15%) while Eastern Sub-Saharan Africa, South Asia and Southeast Asia had the smallest fraction at approximately 5%.

**Figure 7:**
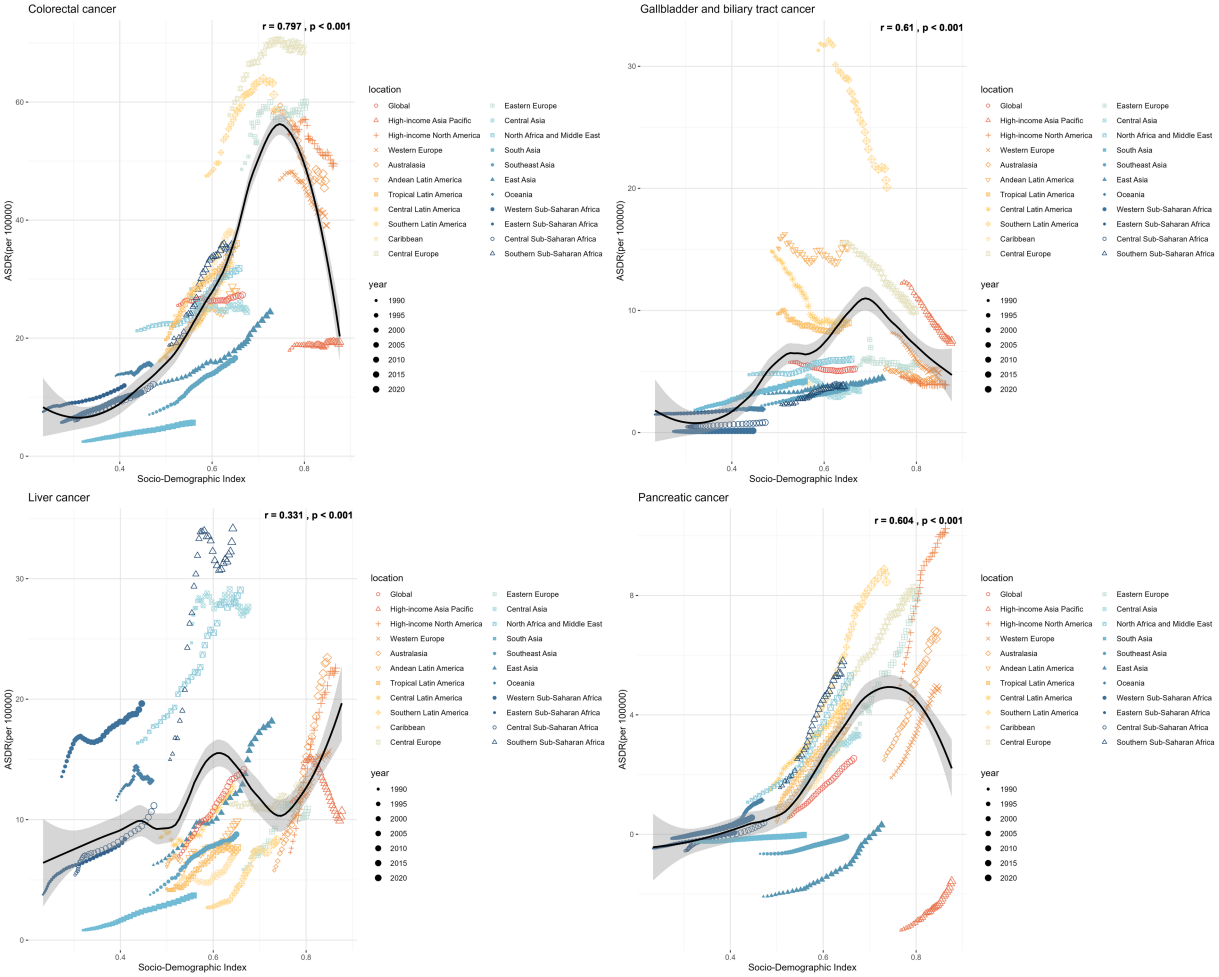
The ASDR of gastrointestinal cancers attributable to high BMI across 204 countries and territories by SDI in 2021.

Hungary, Slovakia, and the Republic of Nauru had the highest ASDR of CRC attributable to high BMI in 2021, with estimated ASDR exceeding 80 per 100,000 population (Figure 7). As SDI increases, the ASDR of CRC increases steadily until SDI reaches around 0.8, which begins to decrease with higher SDI values exceeding 0.8. Austria, San Marino, and Czechia displayed the most significant declines in ASDR trends, while Cabo Verde, Vietnam, and Lesotho exhibited the most substantial increases over the period.

### Liver cancer

In 2021, the regions with the highest ASDR of liver cancer attributed to high Body Mass Index (BMI) were Southern Sub-Saharan Africa (ASMR: 1.26, [0.53–2.11] per 100,000 population; ASDR: 34.16 [14.22–57.28] per 100,000 population), followed by North Africa and the Middle East, Central Asia, Australasia and high-income North America (Supplementary Table S3; Figures 3, 4). In contrast, South Asia, the Caribbean, Tropical Latin America, Southeast Asia and Eastern Sub-Saharan Africa exhibited the lowest ASRs. All regions experienced an increase in the ASRs of liver cancer attributed to high BMI over the past three decades, except for high-income Asia Pacific, with an EAPC of −0.39% in ASMR and −0.89% in ASDR (Figures 5, 6; Supplementary Table S3). South Asia experienced the most rapid increase, with both the ASMR and ASDR of liver cancer attributable to high-BMI increasing 5% annually. East Asia, Australasia, high-income North America and Southern Latin America also witnessed significant increase, with an approximate 4% EAPC for both the ASMRs and ASDRs.

In 2021, Mongolia had the highest ASDR of liver cancer attributed to high BMI, standing at 243.77 per 100,000 population, followed by Tonga and Egypt, both exceeding 100 per 100,000 population (Figure 7). There was no significant change between the ASDR of liver cancer as SDI increases. Some countries, such as Mauritius, Kuwait, and Czechia, witnessed a reduction in the ASDR of liver cancer, whereas Nepal, India, and Australia experienced the most significant increase during the same period.

### Gallbladder and biliary tract cancer

Between 1990 and 2021, Latin America regions exhibited the highest ASRs of GBTCs attributable to high-BMI while Sub-Saharan regions consistently reported the lowest ASRs (Figures 3, 4; Supplementary Table S2). Southern Latin America, the region with the highest ASRs, witnessed a decline of both the ASMR and ASDR, with EAPCs of −1.5% (−1.59% to −1.41%) and −1.53% (−1.64% to −1.41%) respectively (Figures 5, 6; Supplementary Table S2). Central Latin America and Western Europe had the most rapid declining trend of the ASRs, with an annual decreasing rate of more than 1.7%. A total of 12 out of 21 GBD regions reported declining trends with the ASRs. Low SDI and low-middle SDI regions reported lower ASRs compared to higher SDI regions but most of these regions had increasing temporal trends. The most rapid increase was observed in South Asia, with the EAPC of 2.86 (2.79–2.92%) in ASMR and 2.79% (2.73–2.84) in ASDR, followed by the Sub-Saharan regions and Southeast Asia.

An increase in the ASDRs of GBTCs attributable to high BMI was noted until the SDI reached approximately 0.75, with reductions observed after SDI exceeded 0.75 (Figure 7). From 1990 to 2021, Turkmenistan, Guatemala, Australia, Bermuda, Greenland, Germany, Sri Lanka, Israel, Hungary, France, Chile, United States Virgin Islands, Ireland, and Czechia witnessed a reduction in the total percent change of ASDR with more than 50%. Conversely, Cabo Verde, Lesotho, and India experienced the most substantial increases with 318, 166, and 153%, respectively.

### Pancreatic cancer

Throughout the past three decades, all countries with available data exhibited an increasing trend with the ASRs of pancreatic cancer attributable to high BMI (Figures 5, 6; Supplementary Table S4). Andean Latin America had the most rapid increase, with the EAPC of 10.13% (8.41–11.89%) in ASMR and 6.41% (5.82–6.99%) in ASDR respectively, followed by Caribbean, Oceania and Tropical Latin America. By 2021, the highest ASRs were observed among high SDI regions, notably high-income North America, Southern Latin America, Central Europe and Eastern Europe.

In 2021, the United Arab Emirates, Hungary, and Greenland reported the highest ASDRs, with estimated values of 15.04, 12.25, and 12.17 per 100,000 population, respectively (Figure 7). As the SDI increases, the ASDR of pancreatic cancer shows a steady rise until the SDI reaches approximately 0.8, after which it begins to decrease as SDI values exceed 0.8. Over half of the countries and territories reported increasing trends with the ASRs of pancreatic cancer attributable to high-BMI.

## Discussion

Our study provides the most up-to-date investigations regarding the burden of gastrointestinal cancers attributable to high BMI, with analysis of the longitudinal trends and geographical disparities of four major digestive malignancies globally. All GI cancers exhibited significant increases in both deaths and DALYs, with liver cancer showing the most dramatic increase from 955 deaths and 21,952 DALYs in 1990 to 9,215 deaths and 223,368 DALYs in 2021, and pancreatic cancer showing a four-fold increase. Over the three decades, colorectal cancer remains as the leading cause of mortality and morbidity among all GI cancers, with steady age-standardized rates. Liver cancer and pancreatic cancer had dramatic rising age-standardized rates while gallbladder and biliary tract cancers showed an annual declining rate of 0.4% with the age-standardized rates globally. Despite the general rising burden of high BMI attributable GI cancers, there is substantial socio-demographic heterogeneity. High SDI and high-middle countries consistently reported higher age-standardized rates of risk-attributed GI cancer in 2021. The age-standardized rates of colorectal cancer, along with gallbladder and biliary tract cancers tended to decrease or increase at a slower pace in high SDI and high-middle SDI countries compared to low SDI and low-middle SDI countries. On the contrary, the age-standardized rates of risk attributable liver cancer and pancreatic cancer showed rising trends in nearly all regions.

Both males and females had significant increases with high BMI attributable GI cancer burden between 1990 and 2021. This rise was characterized by distinct sex-specific patterns: males consistently demonstrated higher ASMR and ASDR for colorectal cancer and liver cancer, whereas females had higher rates for gallbladder and biliary tract cancer. These disparities are underpinned by a combination of behavioral and biological mechanisms. Epidemiologically, the higher burden of CRC and liver cancer in males is strongly correlated with a greater prevalence of obesity-driven metabolic dysfunction, including non-alcoholic fatty liver disease (NAFLD)—a key precursor to hepatocellular carcinoma—and pro-carcinogenic shifts in the gut microbiome (18, 19). For instance, previous reviews have identified male sex as an independent risk factor for NAFLD progression, even after adjusting for BMI (20). Conversely, the sexual dimorphism in GBTC is largely attributed to hormonal influences. Estrogen, through its effect on cholesterol metabolism and gallbladder motility, significantly increases the risk of cholelithiasis, which is present in the majority of the GBTC cases (21, 22). Furthermore, while estrogen is recognized for its protective role against CRC and liver cancer via anti-inflammatory and insulin-sensitizing pathways, its role in GBTC pathogenesis highlights the complex, tissue-specific nature of sex hormone action (23, 24). Our findings on pancreatic cancer, which show a sex-age interaction, further support the need for nuanced investigation. The higher DALY rates in males under age 60 may reflect a stronger cumulative impact of lifestyle factors, while the reversal in older age groups could point to the loss of protective hormonal effects post-menopause. These observed disparities underscore the critical need for sex-stratified research and the future implementation of sex-specific public health interventions and treatment paradigms.

Gastrointestinal cancers are typically a disease of the elderly population. Our analysis indicated increasing rates of risk-attributed GI cancers as age advances. Substantially higher age-specific DALY rates were documented for all GI cancers among individuals aged 50 years and older. However, recent findings suggest a decline in the incidence rates of several GI cancers in individuals over 50, likely stemming from advancements in early detection, diagnosis, and treatment protocols (25–27). Conversely, a concerning rise in early-onset GI cancers (diagnosed before the age of 50) is being observed globally. For instance, between 2004 and 2016, colorectal cancer incidence increased annually by 7.9, 4.9, and 1.6% among individuals aged 20–29, 30–39, and 40–49 across 20 European nations (28). Similarly, data from 9 high-income countries reported the incidence of colorectal cancer to increase by 0.8–4% per year among those younger than 50 (29). While the underlying causes for this shift are multifactorial, the parallel timelines of the rising trends of early-onset GI cancer and the global obesity epidemic are striking and unlikely to coincidental. Data from the Nurses’ Health Study revealed that increased BMI at age 18 years, and weight gain of 40 kg or more since 18 were associated with increased risk of early-onset CRC (30). Similarly, a large population-based case–control study concluded increased risks for early-onset CRC with high BMI, with estimated odds ratio ranging from 1.9 to 2.6 (31). Additionally, previous research found rising incidence of several early-onset GI cancers aligning with the global obesity epidemic with global and country-level data (32). Although increased screening practices may contribute to the rise of some early-onset cancers, it is insufficient to explain the trend for most digestive malignancies (33). The increasing prevalence of early-onset GI cancers is now largely attributed to the early life exposure to behavioral and metabolic risk factors, with obesity being a primary driver (33, 34). Obesity acts as a potent promotor of carcinogenesis through mechanisms including chronic inflammation, insulin resistance, hyperleptinemia, and alterations in the gut microbiome (33, 34). Furthermore, recent research have proposed novel obesity indices that offer more granular risk assessment to capture the metabolic dysfunction driving carcinogenesis. For example, data from longitudinal cohorts in China and US have shown that obesity indices, such as triglyceride glucose index (TyG), are strongly associated with GI cancer risk (35, 36). Other indices integrating metabolic parameters, such as biomarkers of insulin resistance, dyslipidemia and inflammation are providing powerful predictors for early-onset GI cancers, even in individuals with normal BMI (37). The combined impact of diverse risk factors, coupled with potential epigenetic interactions, gut dysbiosis, and metabolic disruptions, contributes to the onset and progression of distinct GI cancers.

Colorectal cancer had the greatest disease burden of all GI cancers attributable to high BMI, with approximately 100 thousand deaths and 2.4 million DALYs in 2021. In alignment with previous research, we found that the risk-attributed burden of colorectal cancers is positively associated with SDI (38, 39). A similar trend was observed for risk-attributed pancreatic and biliary tract cancers, showcasing increased ASRs in higher SDI countries where obesity prevalence is higher compared to lower SDI nations. Accordingly, GBD reported an age-standardized summary exposure value of 32.55 for high BMI in high SDI regions, contrasting with 12.46 in low SDI regions, indicating a nearly three-fold difference. This variation is likely influenced by lifestyle factors such as sedentary behaviors and consumption of processed foods (38, 39). On the other hand, the age-standardized rate of risk-attributed liver cancer did not show significant association with SDI. Primary liver cancer had different etiological risk factors, including hepatitis C virus (HCV) or hepatitis B virus (HBV) infection, alcohol-related liver disease (ALD) or metabolic dysfunction-associated steatotic liver disease (MASLD) (40, 41). The global burden of liver cancer varies geographically due to variations in the distribution of these risk factors and disparities in screening and treatment accessibility. While Asian and African countries report higher incidence and mortality rates of liver cancer primarily driven by HBV and HCV infections, our results highlight the global public health concern posed by obesity-related liver cancer (41, 42).

Our study also showed that the ASRs of risk-attributed colorectal cancer and biliary tract cancers decreased in higher SDI countries and increased in lower SDI countries, which is likely due to improved diagnosis, treatment and healthcare resources among higher SDI countries (43, 44). These findings align with broader epidemiological shifts in obesity and Westernized lifestyle adoption—historically concentrated in high SDI nations—toward lower SDI regions, where rising ASRs mirror these transitions (45, 46). On the other hand, risk-attributed liver cancer exhibited increasing ASRs across nearly all regions, with the most pronounced rises occurring in both high and low-middle SDI regions. Advances in HBV immunization and antiviral therapies for HBV and HCV have contributed to a shifting etiology of liver cancer, transitioning from viral hepatitis-related cases to non-viral etiologies (41). Data from previous GBD studies indicated rising incidence and mortality rates of MASLD-related liver cancer while HCV and HBV related liver cancer showed declining rends (47, 48). This shift is further underscored by our analysis, which identified a doubling in the proportion of age-standardized DALYs for liver cancer attributable to high BMI—from 6.97% in 1990 to 14.16% in 2021. Notably, projections suggest MASLD-related liver cancer incidence will continue to increase until 2030 (49, 50). We also found that pancreatic cancer showed higher increasing rates in higher SDI countries compared to lower SDI countries. Pancreatic cancer is one of the deadliest cancers, with an overall 5-year survival rate around 5%, and the majority of patients diagnosed at advanced stages (51). The steeper increase in high SDI settings may reflect the growing burden of modifiable risk factors, including tobacco use, alcohol consumption, diabetes, and pancreatitis. Previous GBD studies highlight that high SDI regions exhibit the highest diabetes prevalence, with age-standardized rates in high-income super regions surging by 114.8% over the past three decades (52, 53). Similarly, pancreatitis rates correlate positively with SDI (54). Nevertheless, the drivers of rising pancreatic cancer burden in high SDI contexts warrant further systematic investigation.

Our study emphasizes the urgent need to address the rising burden of high BMI attributed gastrointestinal cancers, especially among resource-constrained settings. To address this challenge, we propose a multi-tiered intervention framework integrating obesity prevention through lifestyle modification, revision of evidence-based screening guidelines, and development of personalized early detection strategies tailored to specific GI malignancies. Lifestyle interventions, which mainly consist of physical activity and dietary changes, could significantly contribute to reducing the burden of obesity-attributed GI cancers (55, 56). Colorectal screening has been proven with cost-effectiveness and recommended by various international guidelines (57). However, emerging risk factors and the rising incidence of early-onset cases necessitate earlier screening initiation and enhanced risk stratification. The American Cancer Society, for instance, now recommends initiating average-risk screening at age 45, with earlier screening for high-risk populations (58). In contrast, cancers of the gallbladder, biliary tract, liver, and pancreas pose unique challenges due to their late-stage diagnosis and the impracticality of universal screening (51, 59). For these malignancies, early detection hinges on improved risk stratification and adoption of novel tools such as blood-based biomarkers, metabolomic profiling, and AI-driven imaging analytics. A randomized controlled trial demonstrated that semi-annual ultrasonography in patients with HBV infection or a history of chronic hepatitis enhanced early liver cancer detection and reduced mortality, illustrating the potential of targeted surveillance (60). Ultimately, curbing the GI cancer burden demands coordinated efforts among policymakers, healthcare providers, and researchers to address modifiable risks, eliminate barriers to screening access, and enhance public awareness among high-risk populations.

There were several limitations with our study. One major limitation was the variations in data quality given the data sources and collections across countries, especially the lack of high-quality data from cancer registries in certain low and low-middle income countries. Further, due to the inherent methodological limitations, we were unable to quantify the disease burden based on the anatomical or histological subtypes of gastrointestinal cancers, such as the anatomical subtypes of colorectal cancer (proximal, distal and rectum) and the major molecular subtypes of liver cancer subtypes (hepatocellular carcinoma or cholangiocarcinoma). Recent research indicated that high-BMI showed limited heterogeneity within the association of high-BMI with colorectal cancer according to either anatomical or molecular subtypes (61, 62). GBD 2021 defined high BMI for adults over 25 kg/m^2^, which is likely to result in biased estimates of risk-attributed gastrointestinal cancer burden due to the heterogeneity of cut-points of high BMI based on different race and ethnicities (63). The universal BMI cut-off value may underestimate the disease burden in certain Asian countries, given their lower BMI cut-off values. Last but not least, our study was unable to capture various confounding factors, including genetic predispositions and potential environmental risk factors.

In conclusion, our comprehensive analysis reveals a rising global burden of gastrointestinal cancers attributable to high BMI. All four GI cancers demonstrated increased mortality and DALYs, with colorectal cancer remaining the largest contributor. Liver and pancreatic cancers showed the most pronounced increases in age-standardized rates. While high SDI and high-middle SDI countries bear the highest ASRs of GI cancers attributable to high-BMI, they have shown declining trends in colorectal cancer, along with gallbladder and biliary tract cancer. On the other hand, nearly all regions have shown increasing trends with the ASRs of risk-attributed liver cancer and pancreatic cancer. These findings underscore the urgent need for multi-level interventions, including lifestyle modifications and tailored screening protocols to address the growing burden of obesity-driven GI cancers.

## Data Availability

The original contributions presented in the study are included in the article/Supplementary material, further inquiries can be directed to the corresponding author.
