# Supplementary material for: The global epidemiology of gastrointestinal cancers attributable to high-BMI: findings from the Global Burden of Disease Study 2021
Source: Front Nutr. 2025 Sep 23;12:1670111. doi: 10.3389/fnut.2025.1670111 (PMC12501786; doi:10.3389/fnut.2025.1670111)
Supplement: Supplementary file 1 [file Table_1.DOCX]

Supplementary Table 1: The age-standardized mortality (ASMR) and DALY (ASDR) rates of colorectal cancers attributable to high BMI across regions in 2021, and the estimated annualized percent change (EAPC) from 1990 to 2021.

|  | ASMR | EAPC in ASMR | ASDR | EAPC in ASDR |
| --- | --- | --- | --- | --- |
| Global | 1.17 (0.51 to 1.87) | 0.00 (-0.04 to 0.04) | 27.33 (11.80 to 43.47) | 0.12 (0.08 to 0.16) |
| Low SDI | 0.32 (0.12 to 0.52) | 1.5 (1.38 to 1.62) | 8,17 (3.23 to 13.20) | 1.33 (1.22 to 1.45) |
| Low-middle SDI | 0.45 (0.19 to 0.71) | 2.72 (2.65 to 2.79) | 11.87 (5.0 to 18.73) | 2.69 (2.63 to 2.76) |
| Middle SDI | 0.82 (0.35 to 1.32) | 2.13 (2.11 to 2.16) | 21.01 (8.93 to 33.45) | 2.10 (2.07 to 2.14) |
| High-middle SDI | 1.67 (0.72 to 2.66) | 0.4 (0.32 to 0.48) | 39.23 (16.94 to 62.34) | 0.31 (0.24 to 0.37) |
| High SDI | 1.68 (0.73 to 2.66) | -0.64 (-0.69 to -0.59) | 40 (17.48 to 62.93) | -0.48 (-0.52 to -0.43) |
| Southeast Asia | 0.62 (0.25 to 1.0) | 3.04 (2.93 to 3.15) | 16.64 (6.73 to 26.92) | 2.84 (2.72 to 2.97) |
| Oceania | 0.59 (0.25 to 0.96) | 0.51 (0.42 to 0.6) | 15.48 (6.43 to 25.08) | 0.44 (0.37 to 0.52) |
| East Asia | 0.96 (0.40 to 1.59) | 2.41 (2.34 to 2.48) | 24.42 (10.09 to 40.83) | 2.31 (2.21 to 2.41) |
| Central Asia | 0.96 (0.41 to 1.53) | 0.1 (-0.03 to 0.22) | 24.46 (10.40 to 38.70) | -0.22 (-0.32 to -0.13) |
| Eastern Europe | 2.53 (1.09 to 4.04) | 0.72 (0.6 to 0.84) | 60 (25.94 to 95.33) | 0.45 (0.31 to 0.58) |
| Central Europe | 3.03 (1.35 to 4.85) | 0.56 (0.42 to 0.69) | 68.94 (30.82 to 110.60) | 0.48 (0.34 to 0.61) |
| Australasia | 2.01 (0.85 to 3.20) | -0.78 (-0.86 to -0.71) | 46.65 (20.18 to 74.39) | -0.94 (-1.02 to -0.85) |
| High Income Asia Pacific | 0.83 (0.32 to 1.31) | 0.32 (0.27 to 0.36) | 19.32 (7.65 to 30.53) | 0.13 (0.09 to 0.18) |
| Western Europe | 1.77 (0.75 to 2.90) | -0.65 (-0.71 to -0.59) | 39.08 (16.77 to 63.51) | -0.67 (-0.74 to -0.6) |
| High-income North America | 1.95 (0.87 to 3.03) | -0.66 (-0.76 to -0.55) | 49.52 (22.63 to 76.52) | -0.42 (-0.5 to -0.43) |
| Southern Latin America | 2.52 (1.12 to 4.11) | 0.9 (0.73 to 1.08) | 58.65 (26.14 to 95.15) | 0.95 (0.8 to 1.11) |
| Caribbean | 1.55 (0.66 to 2.56) | 1.68 (1.62 to 1.73) | 37.94 (16.17 to 62.94) | 1.66 (1.61 to 1.71) |
| Andean Latin America | 1.15 (0.49 to 1.93) | 1.81 (1.69 to 1.94) | 28 (12.28 to 47.21) | 1.67 (1.55 to 1.79) |
| Central Latin America | 1.26 (0.56 to 2.06) | 2.11 (2.04 to 2.19) | 32.79 (14.66 to 52.33) | 2.32 (2.25 to 2.4) |
| Tropical Latin America | 1.42 (0.60 to 2.29) | 1.87 (1.76 to 1.98) | 36.04 (15.20 to 57.63) | 1.89 (1.79 to 2.0) |
| North Africa and Middle East | 1.31 (0.58 to 2.10) | 1.7 (1.54 to 1.85) | 31.83 (13.68 to 50.63) | 1.46 (1.32 to 1.6) |
| South Asia | 0.20 (0.08 to 0.32) | 2.81 (2.76 to 2.86) | 5.67 (2.22 to 8.92) | 2.74 (2.69 to 2.78) |
| Central Sub-Saharan Africa | 0.49 (0.18 to 0.83) | 2.41 (2.24 to 2.57) | 12.19 (4.62 to 21.03) | 2.34 (2.19 to 2.5) |
| Eastern Sub-Saharan Africa | 0.48 (0.18 to 0.80) | 1.62 (1.52 to 1.72) | 11.97 (4.57 to 19.80) | 1.33 (1.23 to 1.43) |
| Western Sub-Saharan Africa | 0.47 (0.19 to 0.75) | 2.39 (2.33 to 2.45) | 10.79 (4.35 to 17.69) | 2.20 (2.14 to 2.25) |
| Southern Sub-Saharan Africa | 1.49 (0.63 to 2.34) | 2.32 (2.06 yo 2.58_ | 35.73 (15.05 to 55.83) | 2.36 (2.1 to 2.62) |

Supplementary Table 2: The age-standardized mortality (ASMR) and DALY (ASDR) rates of gallbladder and biliary tract cancers attributable to high BMI across regions in 2021, and the estimated annualized percent change (EAPC) from 1990 to 2021.

|  | ASMR | EAPC in ASMR | ASDR | EAPC in ASDR |
| --- | --- | --- | --- | --- |
| Global | 0.24 (0.16 to 0.33) | -0.46 (-0.53 to -0.38) | 5.20 (3.56 to 7.17) | -0.44 (-0.52 to -0.37) |
| Low SDI | 0.08 (0.05 to 0.11) | 1.76 (1.69 to 1.83) | 2.00 (1.23 to 2.81) | 1.56 (1.51 to 1.61) |
| Low-middle SDI | 0.17 (0.11 to 0.24) | 1.83 (1.78 to 1.88) | 4.22 (2.73 to 5.75) | 1.82 (1.78 to 1.87) |
| Middle SDI | 0.21 (0.14 to 0.28) | 0.52 (0.45 to 0.59) | 4.84 (3.27 to 6.64) | 0.47 (0.39 to 0.55) |
| High-middle SDI | 0.28 (0.19 to 0.40) | -0.62 (-0.7 to -0.55) | 6.25 (4.15 to 8.78) | -0.68 (-0.75 to -0.61) |
| High SDI | 0.28 (0.18 to 0.39) | -1.15 (-1.25 to -1.06) | 5.76 (3.90 to 7.99) | -1.31 (-1.41 to -1.21) |
| Southeast Asia | 0.15 (0.10 to 0.22) | 1.73 (1.67 to 1.78) | 3.64 (2.29 to 5.14) | 1.48 (1.41 to 1.56) |
| Oceania | 0.07 (0.05 to 0.11) | 0.1 (0.06 to 0.14) | 1.91 (1.14 to 2.76) | 0.04 (0 to 0.08) |
| East Asia | 0.20 (0.11 to 0.29) | 1.18 (1.10 to 1.27) | 4.45 (2.54 to 6.63) | 1.09 (1.02 to 1.17) |
| Central Asia | 0.14 (0.10 to 0.20) | -0.78 (-1.22 to -0.34) | 3.41 (2.30 to 4.71) | -1.0 (-1.43 to -0.57) |
| Eastern Europe | 0.25 (0.17 to 0.34) | -0.12 (-0.38 to 0.14) | 5.68 (3.80 to 7.80) | -0.3 (-0.57 to -0.03) |
| Central Europe | 0.46 (0.31 to 0.64) | -1.6 (-1.67 to -1.53) | 10.03 (6.81 to 13.89) | -1.59 (-1.64 to -1.53) |
| Australasia | 0.20 (0.13 to 0.28) | -0.69 (-0.81 to -0.58) | 4.26 (2.88 to 5.93) | -0.8 (-0.93 to -0.67) |
| High Income Asia Pacific | 0.40 (0.26 to 0.55) | -1.36 (-1.41 to -1.32) | 7.51 (4.97 to 10.30) | -1.77 (-1.83 to -1.72) |
| Western Europe | 0.24 (0.16 to 0.33) | -1.70 (-1.85 to -1.54) | 4.87 (3.29 to 6.71) | -1.79 (-1.94 to -1.64) |
| High-income North America | 0.17 (0.12 to 0.23) | -0.62 (-0.7 to -0.55) | 3.89 (2.65 to 5.25) | -0.53 (-0.62 to -0.44) |
| Southern Latin America | 0.86 (0.58 to 1.20) | -1.5 (-1.59 to -1.41) | 20.09 (13.60 to 27.58) | -1.53 (-1.64 to -1.41) |
| Caribbean | 0.14 (0.09 to 0.19) | -0.97 (-1.09 to -0.84) | 3.36 (2.29 to 4.64) | -0.94 (-1.07 to -0.81) |
| Andean Latin America | 0.64 (0.40 to 0.92) | -0.07 (-0.21 to 0.07) | 15.15 (9.38 to 22.27) | -0.23 (-0.37 to -0.08) |
| Central Latin America | 0.39 (0.26 to 0.54) | -1.86 (-2.04 to -1.68) | 9.33 (6.20 to 13.06) | -1.89 (-2.1 to -1.69) |
| Tropical Latin America | 0.38 (0.25 to 0.52) | -0.41 (-0.54 to -0.28) | 8.98 (6.09 to 12.27) | -0.46 (-0.61 to -0.31) |
| North Africa and Middle East | 0.27 (0.17 to 0.38) | 1.13 (1.02 to 1.25) | 6.01 (3.79 to 8.58) | 0.92 (0.82 to 1.02) |
| South Asia | 0.17 (0.10 to 0.23) | 2.86 (2.79 to 2.92) | 4.18 (2.46 to 5.70) | 2.79 (2.73 to 2.84) |
| Central Sub-Saharan Africa | 0.03 (0.02 to 0.05) | 2.07 (1.92 to 2.21) | 0.82 (0.48 to 1.28) | 2.0 (1.85 to 2.14) |
| Eastern Sub-Saharan Africa | 0.08 (0.05 to 0.12) | 0.91 (0.82 to 1.01) | 1.91 (1.15 to 2.81) | 0.7 (0.6 to 0.8) |
| Western Sub-Saharan Africa | 0.01 (0.00 to 0.01) | 2.41 (2.05 to 2.76) | 0.15 (0.08 to 0.21) | 2.09 (1.74 to 2.44) |
| Southern Sub-Saharan Africa | 0.16 (0.10 to 0.23) | 1.97 (1.79 to 2.15) | 3.80 (2.28 to 5.42) | 1.93 (1.76 to 2.1) |

Supplementary Table 3: The age-standardized mortality (ASMR) and DALY (ASDR) rates of liver cancers attributable to high BMI across regions in 2021, and the estimated annualized percent change (EAPC) from 1990 to 2021.

|  | ASMR | EAPC in ASMR | ASDR | EAPC in ASDR |
| --- | --- | --- | --- | --- |
| Global | 0.53 (0.21 to 0.90) | 2.37 (2.29 to 2.45) | 14.16 (5.77 to 24.06) | 2.31 (2.22 to 2.39) |
| Low SDI | 0.28 (0.11 to 0.49) | 1.35 (1.27 to 1.43) | 8.17 (3.03 to 14.20) | 1.36 (1.28 to 1.44) |
| Low-middle SDI | 0.43 (0.18 to 0.71) | 2.57 (2.47 to 2.67) | 11.68 (5.02 to 19.05) | 2.65 (2.54 to 2.76) |
| Middle SDI | 0.47 (0.19 to 0.80) | 3.03 (2.94 to 3.13) | 13.09 (5.35 to 22.58) | 2.91 (2.81 to 3.0) |
| High-middle SDI | 0.58 (0.23 to 1.03) | 1.99 (1.90 to 2.08) | 16.05 (6.44 to 28.59) | 2.00 (1.91 to 2.09) |
| High SDI | 0.69 (0.28 to 1.13) | 2.52 (2.33 to 2.71) | 17.19 (7.16 to 28.19) | 2.34 (2.13 to 2.54) |
| Southeast Asia | 0.30 (0.12 to 0.52) | 2.88 (2.63 to 3.12) | 8.78 (3.46 to 15.10) | 2.58 (2.31 to 2.85) |
| Oceania | 0.45 (0.18 to 0.80) | 0.43 (0.26 to 0.59) | 13.25 (5.21 to 24.16) | 0.32 (0.14 to 0.49) |
| East Asia | 0.61 (0.24 to 1.08) | 3.87 (3.73 to 4.01) | 18.16 (7.14 to 33.32) | 3.63 (3.48 to 3.78) |
| Central Asia | 1.05 (0.44 to 1.88) | 0.30 (0.18 to 0.41) | 27.65 (11.45 to 49.51) | 0.06 (-0.06 to 0.18) |
| Eastern Europe | 0.42 (0.17 to 0.72) | 2.28 (1.96 to 2.59) | 10.85 (4.40 to 18.23) | 2.15 (1.8 to 2.5) |
| Central Europe | 0.52 (0.21 to 0.91) | 0.48 (0.33 to 0.64) | 12.87 (5.22 to 22.57) | 0.37 (0.22 to 0.52) |
| Australasia | 0.91 (0.38 to 1.54) | 4.81 (4.64 to 4.97) | 23.45 (9.83 to 39.98) | 4.64 (4.50 to 4.78) |
| High Income Asia Pacific | 0.44 (0.18 to 0.74) | -0.39 (-0.82 to 0.05) | 10.72 (4.33 to 17.89) | -0.89 (-1.29 to -0.49) |
| Western Europe | 0.67 (0.;27 to 1.18) | 2.14 (1.99 to 2.29) | 15.68 (6.28 to 27.59) | 2.05 (1.90 to 2.20) |
| High-income North America | 0.88 (0.38 to 1.44) | 3.92 (3.66 to 4.17) | 22.58 (9.85 to 36.53) | 3.80 (3.49 to 4.10) |
| Southern Latin America | 0.34 (0.14 to 0.57) | 4.46 (4.21 to 4.71) | 8.34 (3.41 to 13.99) | 4.30 (4.04 to 4.56) |
| Caribbean | 0.26 (0.11 to 0.44) | 1.03 (0.76 to 1.3) | 6.93 (2.87 to 11.87) | 0.98 (0.71 to 1.25) |
| Andean Latin America | 0.38 (0.16 to 0.68) | 1.72 (1.47 to 1.96) | 9.87 (4.12 to 17.42) | 1.36 (1.11 to 1.61) |
| Central Latin America | 0.52 (0.22 to 0.89) | 1.52 (1.24 to 1.80) | 12.76 (5.42 to 21.21) | 1.38 (1.08 to 1.67) |
| Tropical Latin America | 0.30 (0.12 to 0.51) | 2.73 (2.48 to 2.98) | 7.68 (3.06 to 13.10) | 2.54 (2.28 to 2.79( |
| North Africa and Middle East | 1.12 (0.48 to 1.88) | 2.06 (1.98 to 2.13) | 29.06 (12.36 to 48.32) | 2.00 (1.93 to 2.07) |
| South Asia | 0.13 (0.05 to 0.21) | 5.32 (5.18 to 5.47) | 3.71 (1.46 to 6.18) | 5.28 (5.12 to 5.44) |
| Central Sub-Saharan Africa | 0.41 (0.12 to 1.01) | 2.13 (2.01 to 2.26) | 11.16 (3.25 to 27.09) | 2.03 (1.91 to 2.15) |
| Eastern Sub-Saharan Africa | 0.30 (0.10 to 0.53) | 2.39 (2.26 to 2.51) | 8.40 (2.87 to 14.81) | 2. 32 (2.18 to 2.46) |
| Western Sub-Saharan Africa | 0.73 (0.29 to 1.22) | 1.05 (0.95 to 1.16) | 19.64 (7.72 to 32.91) | 0.89 (0.78 to 1.01) |
| Southern Sub-Saharan Africa | 1.26 (0.53 to 2.11) | 2.46 (1.85 to 3.07) | 34.16 (14.22 to 57.28) | 2.26 (1.63 to 2.90) |

Supplementary Table 4: The age-standardized mortality (ASMR) and DALY (ASDR) rates of pancreatic cancers attributable to high BMI across regions in 2021, and the estimated annualized percent change (EAPC) from 1990 to 2021.

|  | ASMR | EAPC in ASMR | ASDR | EAPC in ASDR |
| --- | --- | --- | --- | --- |
| Global | 0.11 (-0.03 to 0.31) | 4.63 (4.42 to 4.84) | 2.54 (-0.56 to 7.16) | 4.84 (4.6 to 5.09) |
| Low SDI | 0.00 (-0.02 to 0.02) | NA | 0.00 (-0.42 to 0.69) | NA |
| Low-middle SDI | 0.03 (-0.01 to 0.09) | NA | 0.82 (-0.22 to 2.48) | NA |
| Middle SDI | 0.04 (-0.03 to 0.15) | NA | 1.12 (-0.58 to 3.79) | NA |
| High-middle SDI | 0.15 (-0.04 to 0.44) | 4.28 (4.16 to 4.41) | 3.71 (-0.78 to 10.61) | 4.36 (4.21 to 4.77) |
| High SDI | 0.22 (-0.03 to 0.58) | 3.36 (3.19 to 3.53) | 5.41 (-0.44 to 13.84) | 3.36 (3.16 to 3.55) |
| Southeast Asia | 0.00 (-0.04 to 0.04) | NA | -0.08 (-0.95 to 1.35) | NA |
| Oceania | 0.04 (-0.01 to 0.12) | 6.98 (6.18 to 7.78) | 1.14 (-0.25 to 3.25) | 4.78 (4.35 to 5.22) |
| East Asia | 0.00 (-0.07 to 0.13) | NA | 0.31 (-1.55 to 3.48) | NA |
| Central Asia | 0.13 (-0.01 to 0.34) | 2.87 (2.65 to 3.09) | 3.31 (-0.26 to 8.54) | 2.77 (2.55 to 3.01) |
| Eastern Europe | 0.33 (-0.01 to 0.79) | 2.87 (2.73 to 3.02) | 8.12 (-0.22 to 19.91) | 2.70 (2.56 to 2.85) |
| Central Europe | 0.34 (-0.01 to 0.85) | 2.01 (1.90 to 2.13) | 8.24 (-0.29 to 20.14) | 1.88 (1.74 to 2.02) |
| Australasia | 0.29 (-0.01 to 0.71) | 3.70 (3.52 to 3.88) | 6.77 (-0.15 to 15.83) | 3.40 (3.25 to 3.56) |
| High Income Asia Pacific | -0.09 (-0.20 to 0.06) | NA | -1.57 (-3.77 to 1.70) | NA |
| Western Europe | 0.21 (-0.04 to 0.62) | 3.32 (3.14 to 3.49) | 4.90 (-0.76 to 13.74) | 3.14 (2.95 to 3.34) |
| High-income North America | 0.43 (0.00 to 0.97) | 2.57 (2.33 to 2.80) | 10.24 (0.09 to 22.47) | 2.31 (2.08 to 2.54) |
| Southern Latin America | 0.35 (-0.01 to 0.87) | 2.89 (2.65 to 3.12) | 8.45 (-0.19 to 20.42) | 2.67 (2.44 to 2.90) |
| Caribbean | 0.12 (-0.02 to 0.34) | 8.01 (6.84 to 9.19) | 3.21 (-0.31 to 8.70) | 6.33 (5.64 to 7.02) |
| Andean Latin America | 0.14 (-0.02 to 0.38) | 10.13 (8.41 to 11.89) | 3.59 (-0.30 to 9.57) | 6.41 (5.82 to 6.99) |
| Central Latin America | 0.17 (-0.01 to 0.43) | 3.71 (3.41 to 4.01) | 4.46 (-0.11 to 10.90) | 3.28 (2.05 to 3.51) |
| Tropical Latin America | 0.18 (-0.02 to 0.38) | 6.11 (5.60 to 6.62) | 4.40 (-0.36 to 11.22) | 5.07 (4.75 to 5.38) |
| North Africa and Middle East | 0.22 (0.00 to 0.50) | 5.80 (5.69 to 5.91) | 5.31 (0.07 to 12.07) | 5.39 (5.28 to 5.5) |
| South Asia | 0.00 (-0.02 to 0.02) | NA | -0.02 (-0.34 to 0.52) | NA |
| Central Sub-Saharan Africa | 0.01 (-0.02 to 0.07) | NA | 0.39 (-0.50 to -1.82) | NA |
| Eastern Sub-Saharan Africa | 0.00 (-0.02 to 0.04) | NA | 0.06 (-0.53 to 1.00) | NA |
| Western Sub-Saharan Africa | 0.02 (-0.01 to 0.08) | NA | 0.56 (-0.27 to 1.96) | NA |
| Southern Sub-Saharan Africa | 0.24 (0.00 to 0.55) | 4.71 (4.35 to 5.08) | 5.79 (0.00 to 13.37) | 4.44 (4.12 to 4.77) |


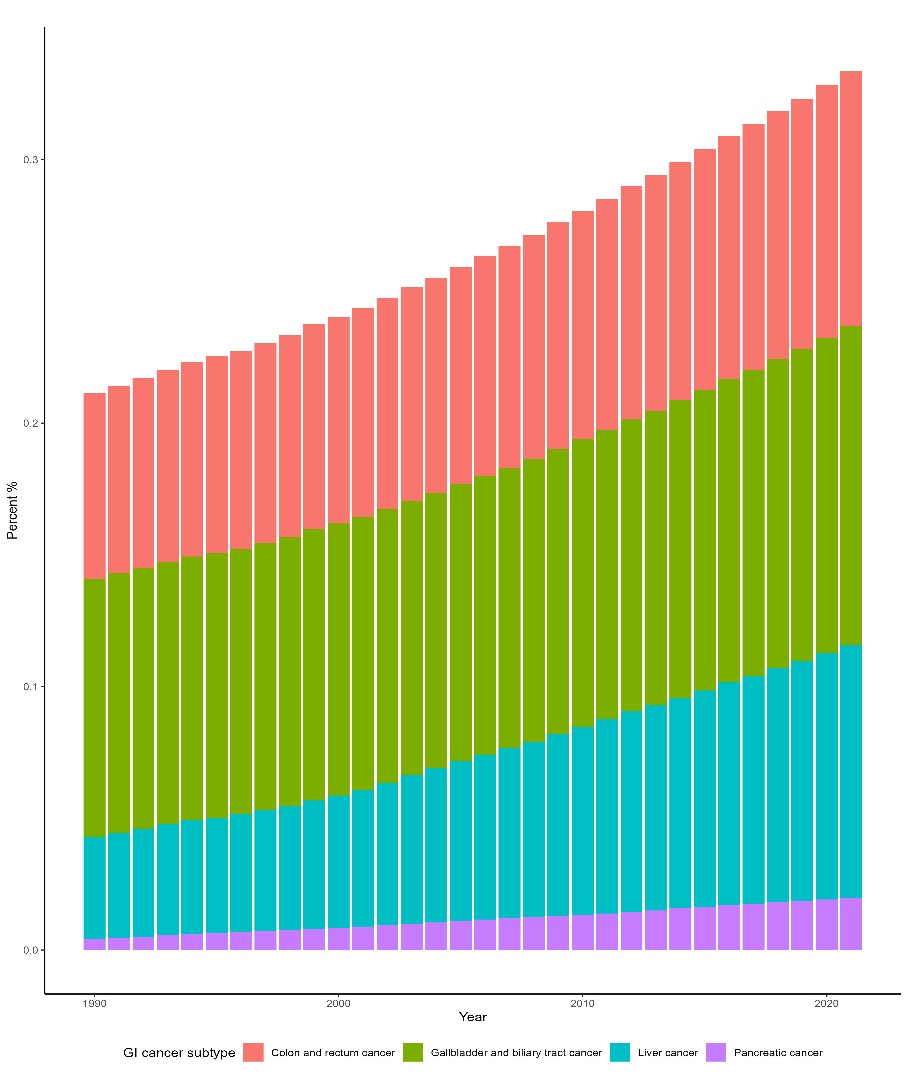


Supplementary Figure 1: The percentage of the age-standardized DALY rates of gastrointestinal cancers attributable to high BMI globally from 1990 to 2021.


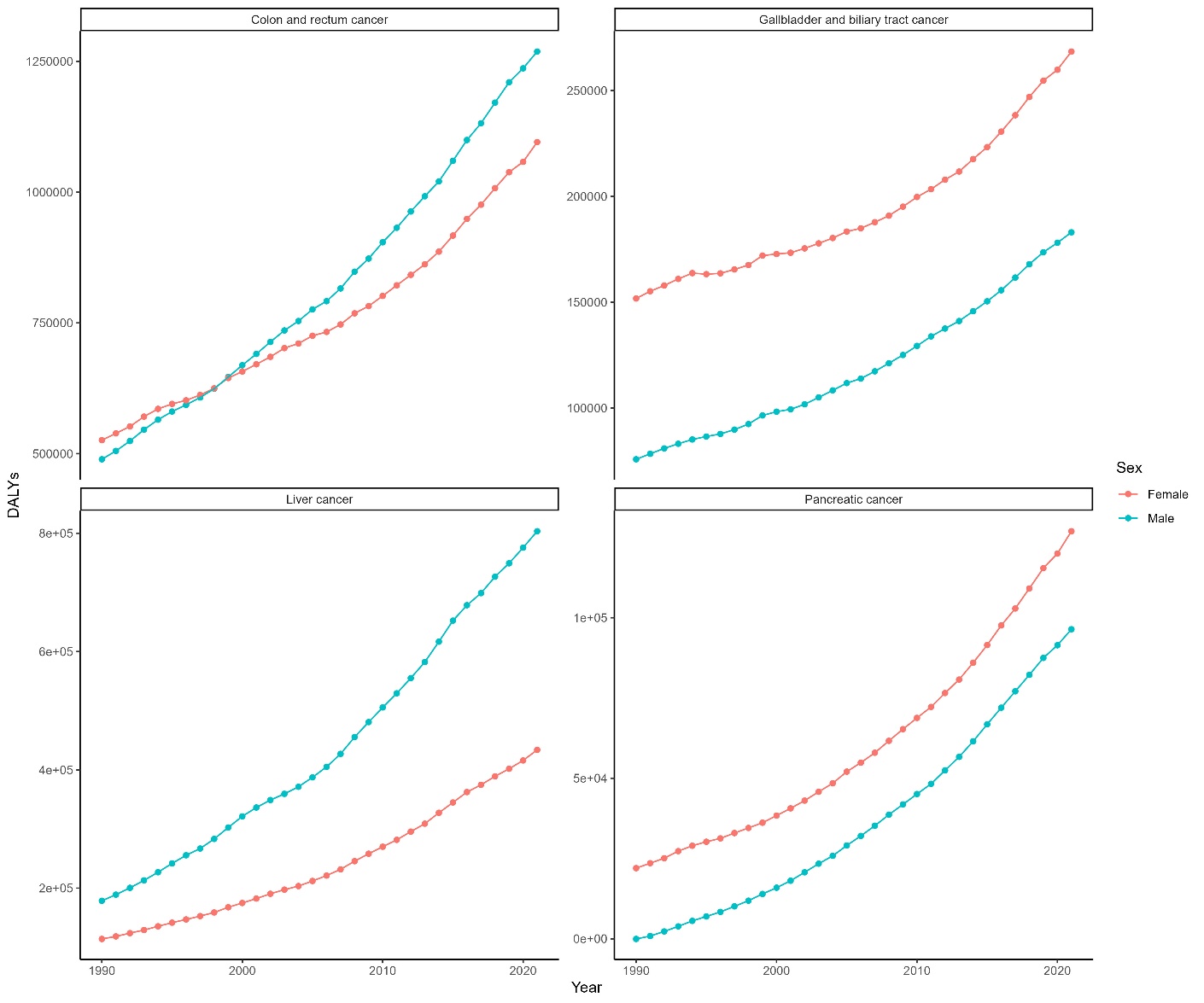


Supplementary Figure 2: The global trend of gastrointestinal deaths attributable to high BMI from 1990 to 2021, stratified by sex.


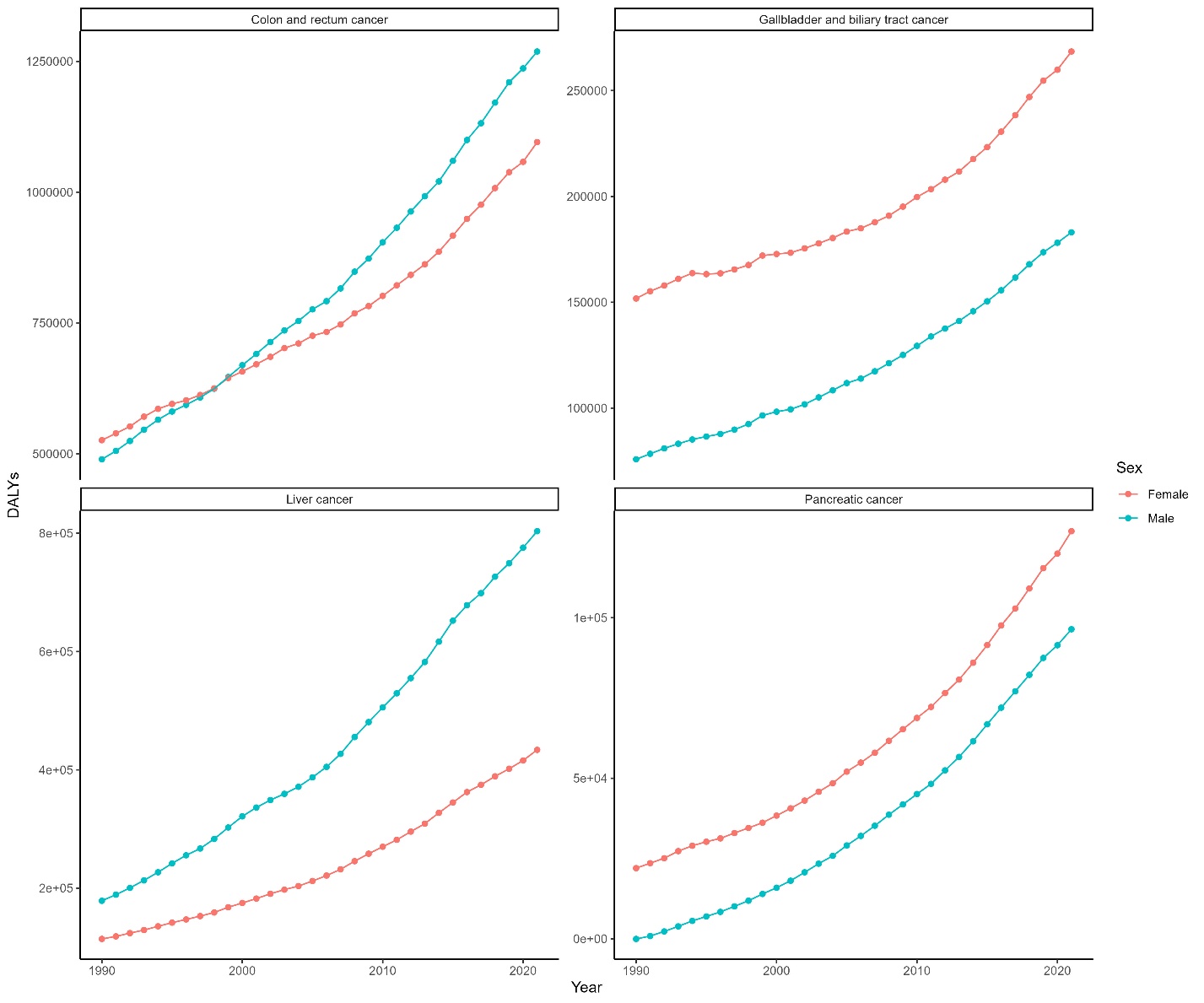


Supplementary Figure 3: The global trend of gastrointestinal DALYs attributable to high BMI from 1990 to 2021, stratified by sex.


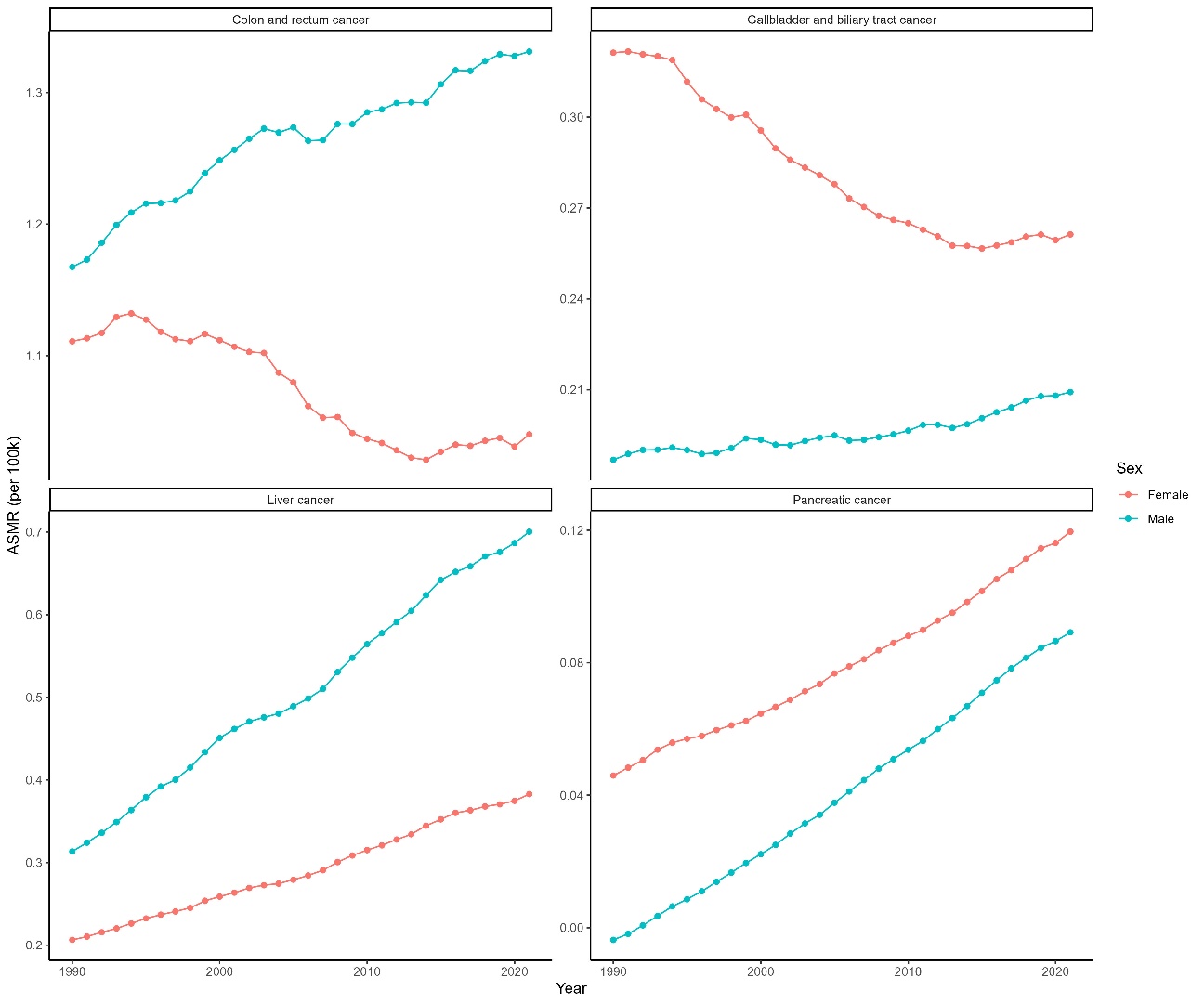


Supplementary Figure 4: The global trend of the age-standardized mortality rate of gastrointestinal attributable to high BMI from 1990 to 2021, stratified by sex.


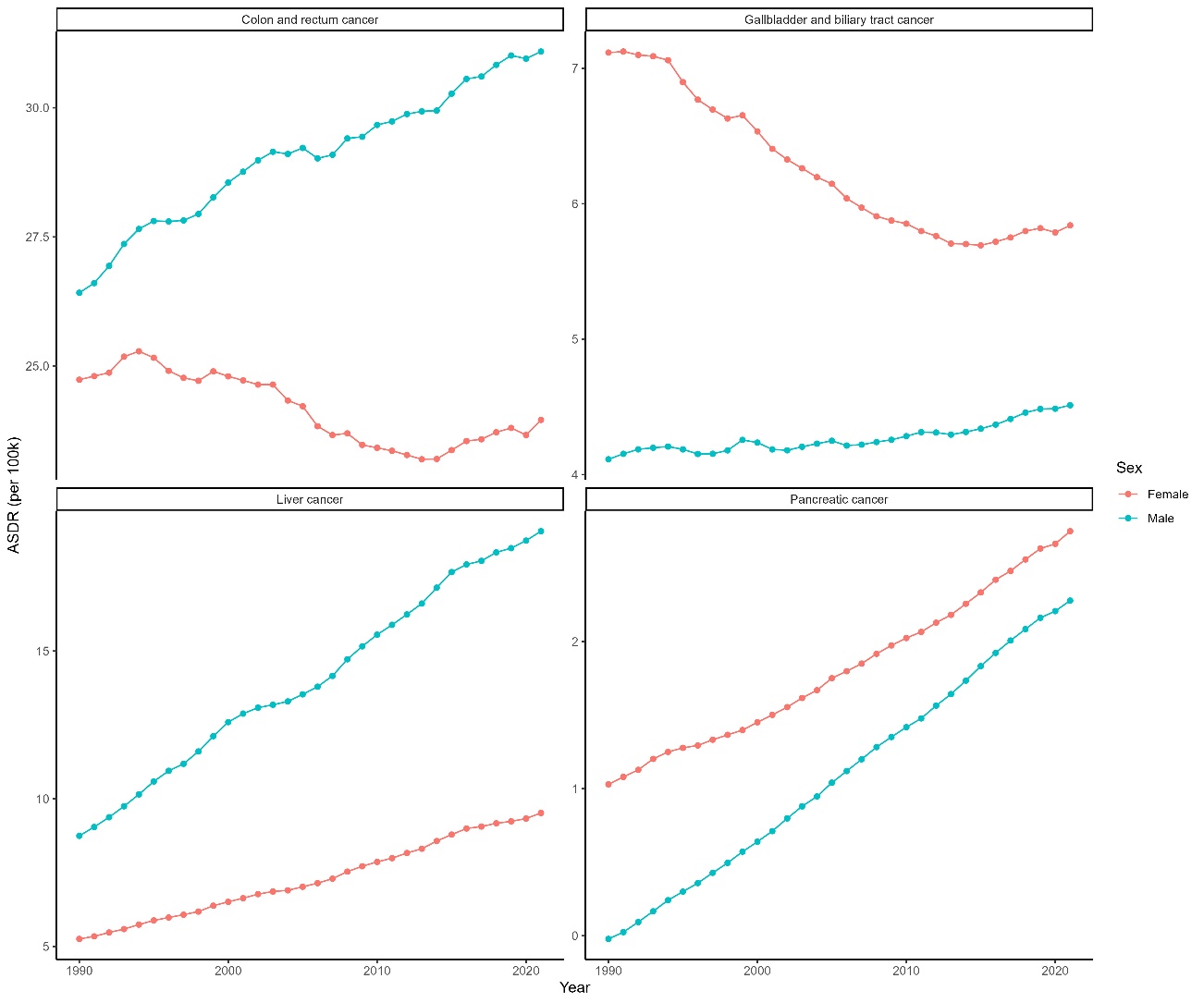


Supplementary Figure 5: The global trend of the age-standardized DALY rate of gastrointestinal attributable to high BMI from 1990 to 2021, stratified by sex.


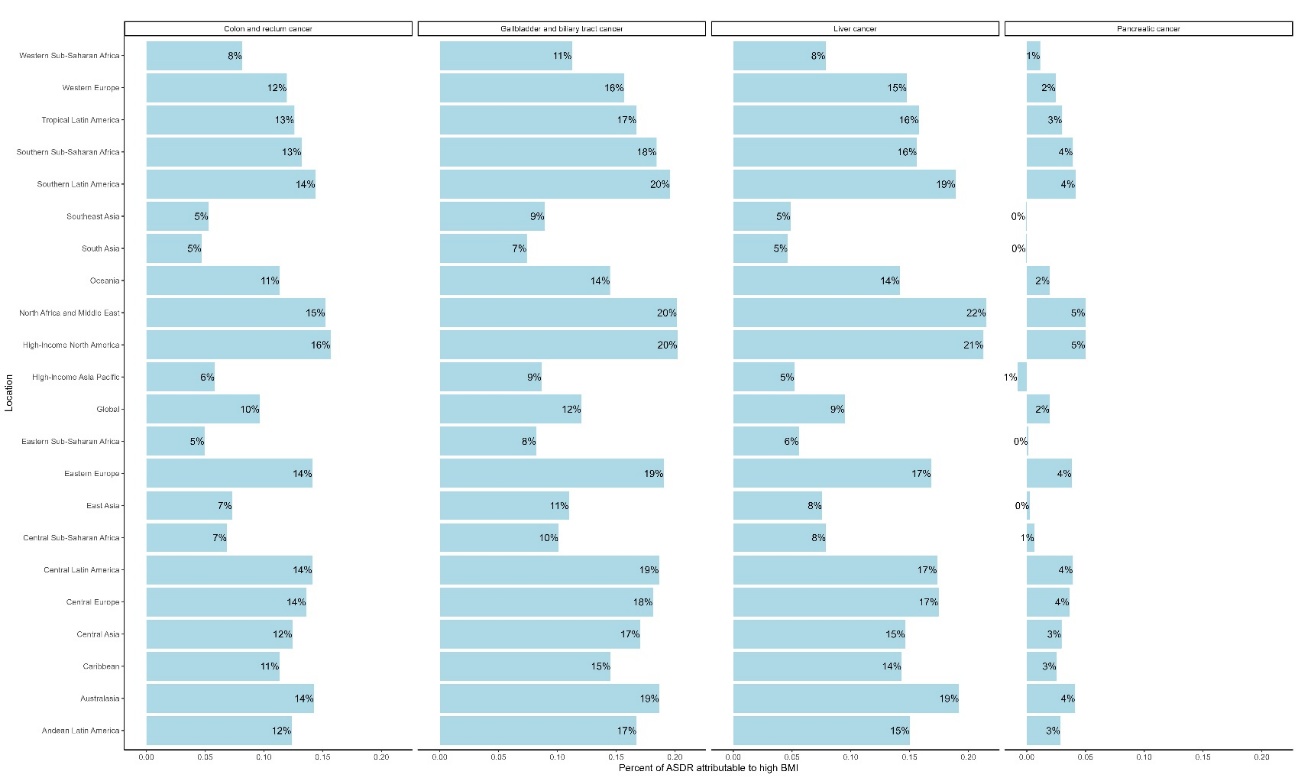


Supplementary Figure 7: The percentage of the age-standardized DALY rates of gastrointestinal cancers attributable to high BMI across GBD regions in 2021.
